# FCOS-LSC: A Novel Model for Green Fruit Detection in a Complex Orchard Environment

**DOI:** 10.34133/plantphenomics.0069

**Published:** 2023-07-19

**Authors:** Ruina Zhao, Yujie Guan, Yuqi Lu, Ze Ji, Xiang Yin, Weikuan Jia

**Affiliations:** ^1^ School of Information Science and Engineering, Shandong Normal University, Jinan 250358, China.; ^2^ School of Engineering, Cardiff University, Cardiff CF24 3AA, UK.; ^3^ School of Agricultural Engineering and Food Science, Shandong University of Technology, Zibo 255000, China.; ^4^ Key Laboratory of Facility Agriculture Measurement and Control Technology and Equipment of Machinery Industry, Zhenjiang 212013, China.

## Abstract

To better address the difficulties in designing green fruit recognition techniques in machine vision systems, a new fruit detection model is proposed. This model is an optimization of the FCOS (full convolution one-stage object detection) algorithm, incorporating LSC (level scales, spaces, channels) attention blocks in the network structure, and named FCOS-LSC. The method achieves efficient recognition and localization of green fruit images affected by overlapping occlusions, lighting conditions, and capture angles. Specifically, the improved feature extraction network ResNet50 with added deformable convolution is used to fully extract green fruit feature information. The feature pyramid network (FPN) is employed to fully fuse low-level detail information and high-level semantic information in a cross-connected and top-down connected way. Next, the attention mechanisms are added to each of the 3 dimensions of scale, space (including the height and width of the feature map), and channel of the generated multiscale feature map to improve the feature perception capability of the network. Finally, the classification and regression subnetworks of the model are applied to predict the fruit category and bounding box. In the classification branch, a new positive and negative sample selection strategy is applied to better distinguish supervised signals by designing weights in the loss function to achieve more accurate fruit detection. The proposed FCOS-LSC model has 38.65M parameters, 38.72G floating point operations, and mean average precision of 63.0% and 75.2% for detecting green apples and green persimmons, respectively. In summary, FCOS-LSC outperforms the state-of-the-art models in terms of precision and complexity to meet the accurate and efficient requirements of green fruit recognition using intelligent agricultural equipment. Correspondingly, FCOS-LSC can be used to improve the robustness and generalization of the green fruit detection models.

## Introduction

With the successful application of artificial intelligence technology in many fields, the development of this modern technology has also stimulated the development of agriculture, making the application of intelligent agriculture in agricultural production more and more extensive. As an important part of automated agricultural intelligent equipment, vision systems have realized practical operations such as fruit picking, yield estimation, fruit counting, and crop type classification in agriculture [1,2]. Intelligent agricultural picking robots can replace or assist manual picking and reduce production costs, so efficient fruit recognition and picking research has received a lot of attention in recent years as an important branch of agricultural robotics [3–6]. Rapid recognition and accurate positioning of fruits in natural scenes can provide key technical support for the machine vision system of fruit-picking robots [7,8].

However, fruit growth in natural environments is characterized by random distribution and mutual occlusion, and fruit images also change dynamically depending on light changes, shooting angles, and distances. In the unstructured agricultural environment, target fruit recognition has become a major challenge for agricultural intelligent devices in production applications [9]. In recent years, fruit detection has been the primary focus of research to identify target fruits from natural environmental backgrounds [10]. Fruit recognition methods mainly include traditional recognition methods based on manual features and deep learning methods for automatic feature extraction.

Traditional fruit recognition algorithms mainly extract information such as color, geometric shape, and texture features of targets and then classify and detect fruiting targets on the basis of machine learning methods. Arefi et al. [11] selected ripe tomatoes in a greenhouse environment for their experimental study and successively processed background informational and color information to finally achieve 96.36% detection accuracy, and the method showed excellent detection only for cases where the fruit color was more clearly distinguished from the background. When the fruit target is close to the background color features, the shape and texture features between the fruit and the background are needed to determine the target region.

Kurtulmus et al. [12] combined color, shape, and texture features using 3 different scales of moving windows to scan unripe green citrus images, and the results of multiple voting window classifiers resulted in a final correct detection rate of 75.3%. Jia et al. [13] segmented the collected apple images under Lab color space using the K-means clustering algorithm and inputted the extracted image red green blue (RGB) and hue saturation intensity color features and geometric shape features into a neural network for fruit recognition, achieving 96.17% fruit recognition accuracy, but the algorithm was relatively tedious to recognize over. Tian et al. [14] proposed a combination of depth images and RGB images to recognize apple fruits by locating the center of the target fruit using depth images and by segmenting it using RGB images with a final recognition efficiency of 96.61%, but the performance of this method was rather poor when dealing with overlapping fruits. Ji et al. [15] proposed an apple recognition and classification algorithm based on a support vector machine with a recognition success rate of 89%, but the algorithm was less effective in detecting fruit with branch and leaf occlusions. Moallem et al. [16] applied K-means clustering and multilayer perceptron to extract apple texture and geometric features and achieved 92.5% and 89.2% classification accuracy.

The above traditional fruit recognition algorithm often involves a series of complicated operations, such as image preprocessing, feature selection, and extraction, which affects the recognition accuracy and speed of the algorithm and makes it difficult to meet the requirements of real-time operation of intelligent devices. Especially when the fruit is close to the background color, a large number of overlapping blocks lead to the inconspicuous shape of the fruit contour, while the change of lighting conditions also leads to the loss of texture features, which seriously interferes with the recognition effect of the algorithm.

With the rapid development of convolutional neural network (CNN), the end-to-end detection process and the advantage of automatic extraction of depth features have reduced many complex operational steps in traditional algorithms. On the basis of this, numerous deep learning-based recognition algorithms such as Faster R-CNN [17], YOLO [18], SDD [19], YOLOv5, FoveaBox [20], and many other mainstream algorithms have been developed, which are far more robust and accurate than traditional recognition algorithms and have been widely used in the field of fruit image detection and segmentation [21–23]. Zhang et al. [24] replaced the original feature extraction network of Faster R-CNN with VGG19 through pretraining network migration and improved the region proposal network structure of the network to improve the detection accuracy of the model for apple fruit and reduce the false detection rate.

Tu et al. [25] designed the model to fuse image color and depth image information with the help of an RGB-D camera and finally achieved 90.9% F1 score to effectively improve the detection accuracy of small target passion fruit. Liang et al. [26] first performed a series of data augmentation techniques to optimize the operation of the data, then redesigned the single shot multibox detector detection frame shape according to the processed dataset, and finally implemented a mango detection model with better performance than Faster R-CNN. Bresilla et al. [27] detected fruits on trees on the basis of an optimized YOLO model, which achieved 90% fruit detection precision by reducing convolutional and pooling layers to make the model shallower and to increase the speed without decreasing the detection precision. Wang et al. proposed a lightweight deep learning model YOLOv5s on the basis of channel pruning, which achieved accurate apple fruit detection with 95.8% detection precision [28].

The above methods require feature area selection based on anchors, requiring the design of anchors of various scales and shapes, and the setting of parameters such as scale, aspect ratio, and number of anchor frames also affects the detection performance of the model. To overcome the drawbacks of anchor-based algorithms, anchor-free algorithms are emerging. Jia et al. [29] used EfficientNetV2-S backbone and a bidirectional weighted feature pyramid network (FPN) as the backbone network for feature extraction, and they used an adaptive training sample selection method to directly select positive and negative samples to obtain higher recall for green fruits at different scales, with detection precision of 62.3%. To eliminate the limitation of the anchor boxes on the model in terms of speed and generalization ability situation, Jia et al. [30] embedded the position attention module in FoveaBox and MaskIoUhead mask calibration module, achieving efficient green fruit recognition.

Considering the problems of the anchor-based methods such as long training time and complicated calculation, the effective and accurate fruit detection model (full convolutional one-stage object detection) algorithm based on LSC attention blocks (FCOS-LSC) is proposed to recognize green fruit by improving anchor-free FCOS [31] as the base model. Instead of normal convolution operation, a deformation convolution [32] is adopted in the backbone network to better extract the fruit features with different shapes. In addition, attention operations [33] are introduced into the multiscale features on scale, space, and channel dimensions to enhance the feature representation of the network. In the classification branch of the detection head, a new positive and negative sample selection strategy is employed to set loss weights for both positive and negative samples to better distinguish between positive and negative samples [34]. The method provides more discriminable supervisory signals and enhances the detection of foreground targets and background environments.

In general, this study has at least the following contributions:

1. In the backbone network, deformable convolution is introduced to better adapt to different fruit shape features during detection.
2. In the neck network, the LSC attention module is embedded in the 3 dimensions of scale, space, and channel of the feature map, which suppress the noise interference in the feature map and make the model focus more on the effective pixel information.
3. In the detection head, a new positive and negative sample determination method is designed to improve the discriminative ability for supervised signals.
4. The proposed method outperforms other advanced methods in terms of accuracy and robustness, which is more suitable for detecting green fruits in complex orchards.

The rest of this paper is organized as follows: Materials and Methods presents the green fruit dataset including image acquisition and dataset production. Next, this section illustrates the proposed FCOS-LSC model including the backbone network, the feature fusion network, and various parts of the detection head, as well as the details of optimization. In Results, experiments are conducted to compare other advanced detection models from different aspects to validate the effectiveness of FCOS-LSC in green fruit detection. Finally, Conclusion summarizes the proposed model and presents future research directions.

## Materials and Methods

### Dataset

There are many disturbances in the complex orchard environment that affect the detection of the vision system, making it difficult for fruit-harvesting robots to recognize green fruits from similarly colored green backgrounds. To better cope with the complexity of the detection task, this study collects and produces 2 green fruit datasets from actual orchards for the experiments, including green apples and green persimmons.

#### Data acquisition

Collection locations: Apple images are collected from the apple production base in Fushan District, Yantai City, Shandong Province, and persimmon images are collected from the back mountain of Shandong Normal University.

Image acquisition equipment: All images are taken with the same camera, Sony Alpha 7II. A total of 1,361 images of green apples and 553 images of green persimmons are taken at a resolution of 6,000 × 4,000 and stored in JPG format.

Acquisition environment: To get closer to the working conditions of the picking robot, fruits in different lighting conditions and different periods are selected as far as possible when capturing images.

**Fig. 1.**
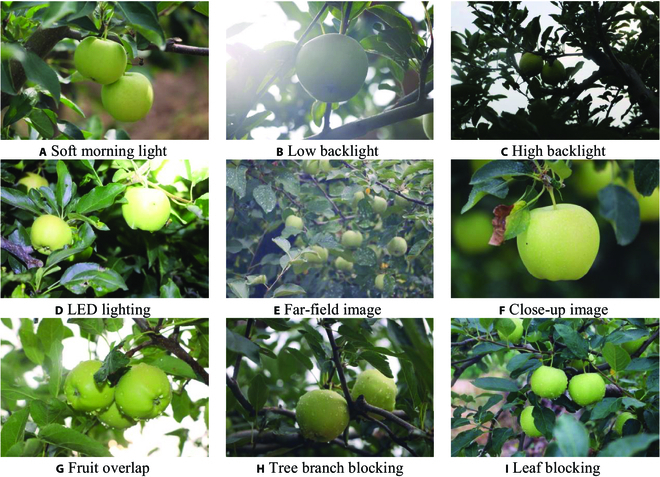
(A to I) Green apple images in different scenes. LED, light-emitting diode.

Shown in Fig. 1A to D, the images of fruits are captured under soft light in the early morning, strong light at noon (which includes images of fruits under low backlight and high backlight conditions), and light-emitting diode lighting at night. As shown in Fig. 1E and F, images from different angles of distant and close views and different directions are captured to imitate the operation of the robot in actual orchards. There are many occlusions and overlaps in the image, including fruit overlapping each other and branch and leaf occlusions. The specific fruit images are listed in Fig. 1G to H.

#### Dataset production

The collected images take full account of the complexities of the orchard, with a certain degree of randomness and representation, and are as close as possible to the requirements of the real-time operation of the machine and equipment. In this paper, the captured images are compressed and scaled to a size of 600 × 400 pixels to enable the fruit detection network to better adapt to the detection requirements of machine equipment for low-resolution images.

LabelMe [35] software was used to annotate the information on green fruits, and corresponding category labels and annotation points are generated and uniformly saved in JSON files. Finally, datasets are generated according to Microsoft COCO [36] format.

Datasets are divided into the training set and validation set according to the ratio of 7:3, in which the training set contains 953 images and the validation set contains 408 images in the apple dataset. The persimmon dataset contains 388 images in the training set and 165 images in the validation set.

### FCOS-LSC detection network

**Fig. 2.**
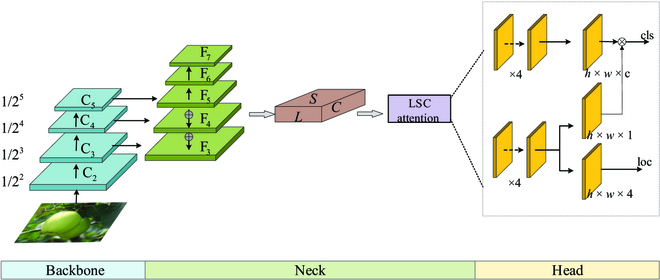
The overview of FCOS-LSC.

FCOS-LSC is an optimization method based on the one-stage object detection model FCOS. The overall framework of the FCOS-LSC model includes the backbone network for feature extraction, the feature fusion structure, the attention module of each dimension on the feature map, and the detection head. The detection head also includes subnetworks for processing classification, bounding box regression, and center point detection. As shown in Fig. 2, the optimized ResNet50 with the addition of deformable convolutional structures is used as the backbone network to improve the feature extraction capability of the network. FPN is utilized to fully fuse the extracted multiscale fruit features. Before input to the detection head, the attention mechanisms are added to the scale, space, and channel dimensions of the feature map by the convolution-based method, which helps the feature map to distinguish foreground fruit objects and background information more effectively. In the detection head, a new label assignment strategy is designed to distinguish between positive and negative samples, providing the detector with a more discriminative supervised signal.

#### Feature extraction and fusion network

As a feature extraction network, ResNet50 performs a series of convolution downsampling operations on the input fruit image to extract fruit feature information. The residual structure solves the problem of gradient disappearance, explosion, and degradation caused by deeper network layers by completely mapping shallow features into deeper networks.

However, in the feature extraction network, the convolution kernel is set to a fixed shape. Fruit feature maps are also limited to extracting valid information only in rectangular filters. The efficiency of fruit detection in complex orchard backgrounds is greatly reduced under uncontrollable conditions such as shooting angles and fruit growth forms, and the design of convolutional kernels with dynamically transformable shapes can adapt to targets with variable morphology and improve recognition accuracy. Therefore, the deformable convolution [32] is added to the C_3_, C_4_, and C_5_ layers of the ResNet50 backbone network to improve feature extraction performance.

**Fig. 3.**
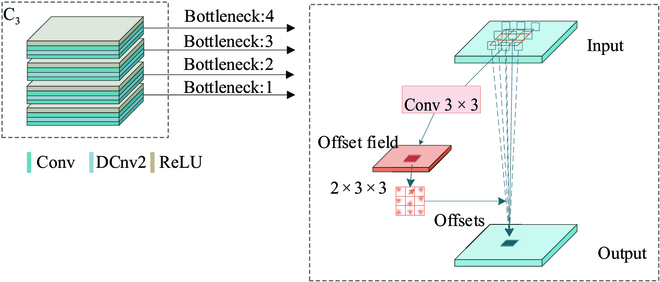
Partially deformable convolutional structures in C_3_ are shown.

The deformable convolution structure is shown in Fig. 3. The size of the convolution kernel is set to 3 × 3, and the same padding as the normal convolution is used to ensure that the size of the output feature map is the same as the size of the input feature map. The 2 × 3 × 3 shift offset values in the convolution kernel correspond to the (*x*, *y*) offset values of each pixel in the 3 × 3 convolution kernel, respectively.

To achieve greater degrees of freedom, different from deformable convolutional networks [37], a learning coefficient of sampling points is added to it, and the coefficient of some useless background sampling points in fruit detection can be set to 0. This coefficient indicates that it has different weights for different pixel values, which improves the feature expression capability. The feature output dimension of the *i*th stage is mapped to 1/2*^i^* of the input image. There are usually many layers that produce output maps of the same size. In this paper, the network layers with the same mapping size are grouped into the same stage layer. After each stage of the ResNet50 layer {C_2_, C_3_, C_4_, C_5_}, the output feature map is mapped to the input image as {1/2^2^, 1/2^3^, 1/2^4^, 1/2^5^}, respectively, as shown in the backbone part of Fig. 2.

The output of the ResNet50 network is a relatively high-level feature map with high semantic information. However, the feature maps after a series of convolution and pooling operations have low resolution. The mappable features are easy to lose details such as boundaries when detecting small objects. FPN realizes the fusion of low-level detail information and high-level semantic information to solve the problem of multiscale prediction. The feature maps C_3_, C_4_, and C_5_ output by the last 3 layers after the ResNet50 network are horizontally connected to the FPN through 1 × 1 convolution. Then, the feature maps perform a 2-fold up-sampling and top-down method to fuse the information of each layer by element addition to obtain F_3_, F_4_, and F_5_. The F_6_ and F_7_ are obtained from F_5_ by 2 convolution operations with a convolution kernel of 3 × 3 and a step of 2, as shown in Fig. 2, Neck section.

#### LSC attention module

**Fig. 4.**
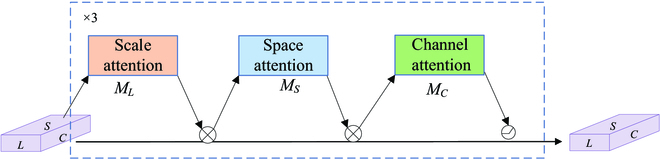
Convolution-based implementation with attention mechanism on each dimension of the feature map. The implementation details of the 3 submodules are shown in Fig. 5.

To enhance the representational capability of the model, an attention learning module implemented by a convolution-based approach is added to the output of the feature fusion network. The LSC attention module is embedded behind the FPN to extract more feature information. The structure is shown in Fig. 2, and the specific implementation is shown in Fig.4.

In this paper, the feature maps output from the FPN are up-sampling and down-sampling to process the high feature layer and low feature layer map scales and adjust to the scale size of the middle feature layer. The feature map can be converted to a 4-dimensional tensor *X* ∈ *R^L×H×W×C^*, redefining *S = H × W*, where *L* denotes the feature level, *H* denotes the feature map height, *W* denotes the feature map width, and *C* denotes the feature channel. The 3-dimensional representation of the feature map is *X* ∈ *R^L×S×C^*. Focusing on the role of the 3 dimensions of the feature map separately can help improve the model feature extraction.

Figure 4 shows the attention operations implemented in the convolution-based feature maps. First, feature maps improve the relationship between fruit scale size differences and features at different levels by operating on the scale dimension. Scale attention can improve the representation ability of feature maps at different levels, thereby improving the perception ability of fruit scale features. Then, through the operation in the spatial dimension, the spatial location information of fruit detection with different geometric shapes is extracted to improve the spatial perception ability of fruit detection. The last part of the cascade operation is the feature channel. The multitasking of fruit detection and segmentation and target representation correspond to features on different channels. Improving the representation learning ability on the feature channel can effectively improve the perceptual ability of the fruit detection task. Finally, the processing in the 3 dimensions is concatenated and multiplied as the input of the detection head. The operation of the attention module in 3 dimensions is as follows.

$$X^{\prime }=M_{L}\left(X\right)⊗X$$ (1)

$$X^{\prime \prime }=M_{S}\left(X^{\prime }\right)⊗X^{\prime }$$ (2)

$$X^{\prime \prime \prime }=M_{C}\left(X^{\prime \prime }\right)⊗X^{\prime \prime }$$ (3)

**Fig. 5.**
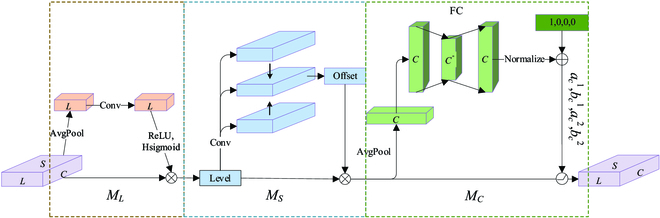
Implementation details of each dimension of the feature map.

As shown in Fig. 5, the *M_L_* module is operated on the scale. The input feature map undergoes a global averaging pooling operation to compress the spatial and channel features into a real number with a global sensory field on space and channel. The linear function is approximated with a 1×1 convolution to generate weights for the feature layer scale by computation. Then, a linear rectification function (ReLU) is used to obtain the nonlinear relationship, which can fit the complex correlation between spatial channels. Finally, the approximate sigmoid is simulated by a hard-sigmoid activation function, which can also shorten the calculation time. The formula is expressed as follows.

$$M_{L}\left(X\right)⊗X=\sigma \left(f\left({\text{Global}}_{{\text{AvgPool}}_{L}}\left(X\right)\right)\right)⊗X$$ (4)

where the *f*(⋅) linear function represents the 1 × 1 convolution and *σ*(⋅) is the hard-sigmoid activation function.

With the understanding of the importance of different semantic information between feature layers in the perception module, the feature map focuses more on the information of each spatial location of the fruit. This includes distinguishing areas where the spatial location of each target in the feature map coexists consistently with the feature layer. Considering the high dimensionality of the spatial dimension, we use deformed convolution to make the spatial feature awareness learning more sparse and then aggregate the features at the same spatial location across feature levels. Figure 5 illustrates the operation process of the *M_S_* module in space. The feature map input to this module is subjected to the convolution kernel size of 3 × 3 deformation convolution to learn the offset and mask information of the spatial position. The feature map focuses on the spatial position of the intermediate layer that is not adjusted at this time and propagates the obtained information to other adjacent feature layers that need to be adjusted in subsequent executions. The input feature map adapts to the same size by performing up-sampling and down-sampling operations on its neighboring feature maps according to the feature level. Aggregate features across layers by simple summation and spatial attention can be learned to obtain more accurate information about offsets at spatial locations. Feature map F_7_, as the top layer, only carries out the up-sampling operation, while F_3_, as the lowest layer, only carries out the down-sampling operation. The other remaining feature layers are scaled accordingly according to the layer determination. The spatial attention formula is expressed as follows.

$$\begin{array}{c} M_{S}\left(X^{\prime }\right)⊗X^{\prime }=\frac{1}{L}\sum_{l=1}^{L}X^{\prime }\\ \text{where}\;X^{\prime }=\sum_{n=1}^{N}W_{n}^{l}X^{\prime }\left(l\;;\;p_{n}+△p_{n};\;c\right)△m_{n} \end{array}$$ (5)

Deformation convolution operation *X*^′^(*l*;  *p_n_* +  △ *p_n_*;  *c*) △ *m_n_* focuses on the *p_n_* +  △ *p_n_* position information of the *c*th channel of the *l*th layer feature, and the self-learning spatial offset is △*p_n_* used to move the position to focus on more obvious areas. The network learns to gain spatial weights $W_{n}^{l}$. We set learnable confidence weights for more important locations △*m_n_*.

The Dynamic ReLU [38] is applied in the *M_C_* module shown in Fig. 5 to execute the feature channel dimensionality awareness module. The input feature map is subjected to global average pooling to compress features in space and scale, and 2 fully connected layers perform channel dimension compression and restoration. The full connection operation is used to predict the importance of each channel and to understand the importance of different channels. The last normalization layer restricts the output to be between [−1, 1]. The calculated weight values of each channel are multiplied by the 2-dimensional matrix of the corresponding channel in the input feature map to realize the weighting of its channel. The weighted feature is added to the input to obtain the output. Feature maps can be shared across spatial channels, channel-shared across spaces. Finally, the input parameters are used to filter the features through the function as the output features.

$$M_{C}\left(X^{\prime \prime }\right)⊗X^{\prime \prime }=\max\left(a_{c}^{1}\left(X^{\prime \prime }\right)X^{\prime \prime }+b_{c}^{1},a_{c}^{2}\left(X^{\prime \prime }\right)X^{\prime \prime }+b_{c}^{2}\right)$$ (6)

$\left[a_{c}^{1},b_{c}^{1},a_{c}^{2},b_{c}^{2}\right]=\theta (\cdot )$ is a hyperfunction that learns to control the activation threshold.

#### Detection head for green fruit prediction

As shown in the head section of Fig. 2, the FCOS-LSC detector consists of task-specific subnetworks. The 3 subnetwork modules of classification, regression, and center point detection constitute the detector part. The classification subnetwork performs the prediction of the confidence that each pixel on the feature map belongs to an apple or a persimmon. Moreover, the regression subnetwork predicts the distance to the 4 edges of the real bounding box of the fruit. The center point detection subnetwork predicts the offset from the target center and shares a portion of the network parameters with the regression network, while the classification network module as a separate network does not share network parameters. Therefore, we use 2 different full convolutional channels to perform specific prediction tasks by decoupling the classification subnetwork and regression molecular network tasks.

The classification subnetwork processes each feature level output from the model Neck structure, and all feature maps share the parameters of the classification subnetwork. The module has 4 convolutional layers with kernel size 3 × 3 and one convolutional layer that performs the prediction of fruit confidence. The bounding box regression subnetwork and the center point detection subnetwork share a part of the fully convolutional network. The full convolution of this part implements 4 convolution layers in parallel with the classification subnetwork, with the size of the convolution kernel 3 × 3. Finally, two 3 × 3 convolution branches are used to output the prediction results of the feature map bounding box and the predicted offset from the center point. The predicted offset is multiplied by the category predicted by the classification subnetwork to output the final confidence score.

Positive and negative sample determination: The original FCOS model assigns positive and negative samples in such a way that the center of the real object bounding box is the center of the circle, and the positive sample area is delimited by a fixed radius. According to the step of feature level, the pixels on the feature map are converted to the coordinates of the corresponding perceptual field region on the input image to directly determine whether the coordinates fall within the divided region. If it falls within the divided region, then it is considered as a positive sample; otherwise, it is a negative sample. When training the sample loss weights, the weights of negative samples are simply obtained from the weights of positive samples, resulting in no new supervisory information provided to the negative sample weights, which limits the detection performance. The proposed method provides more signal discriminative supervision to the detector from different perspectives by specifying the loss weights of positive and negative samples.

The built positive sample weighting function takes the predicted fruit category confidence and the intersection over union (IoU) between the predicted box and the ground truth as input. This paper sets positive sample weight by estimating the degree of agreement between the class network and the regression network. The negative sample weight function takes the same input as the positive sample weight function, but the negative sample weight is represented by the product of the probability that the anchor frame is a negative sample and its importance if it is a negative sample. The fuzzy prediction frame with the same positive sample weight can get a finer supervision signal because of the different negative sample weights.

First, this paper constructs a set of candidate positive samples by selecting the detection box near the center point of the ground-truth bounding box. During testing, all predictions for the fruit category are appropriately ranked by a combination of a confidence score and the predicted IoU as a ranking metric to rank detection boxes in the candidate set. The correctness of each prediction box is checked from the beginning of the ranked list. Highly ranked fruit category prediction scores and high IoU are sufficient requisites for positive prediction. Positive sample weights are positively correlated with prediction scores and IoU. Therefore, the positive sample weighting function is defined as follows.

$$\begin{array}{l} w_{\mathrm{pos}}=e^{\mu \times s\times {\mathrm{IoU}}^{\beta }}\times s\times {\mathrm{IoU}}^{\beta }\\ \;\text{where}=\;\mathrm{IoU}\frac{\text{Intersection}\left(b,b^{\prime }\right)}{\text{Union}\left(b,b^{\prime }\right)} \end{array}$$ (7)

where *s* is the category score of the predicted fruit, and *b*, *b^′^* are the positions of the predicted box and the ground box. The *s ×* IoU*^β^* can indicate the degree of agreement between the predictions of the classification network and the regression network in forward prediction. The *β* is used as a balancing factor. The exponential function is used to enhance the variance of positive sample weights. The *μ* is used as a hyperparameter to control the relative gap between different positive sample weights. Positive sample weight can emphasize that consistent boxes have higher classification scores and higher IoU, but inconsistent boxes cannot be distinguished by positive sample weight. According to the IoU, to determine whether the detection box is incorrectly predicted, the IoU smaller than the threshold is the only factor to determine the negative sample probability denoted by *P*_neg_.

The interval is divided into [0.5,0.95] according to the evaluation index of the COCO data format. When the IoU is less than 0.5, the lower limit of the evaluation interval, the probability of a negative sample is 1. When the IoU is greater than the upper limit of the evaluation interval, the probability of a negative sample is 0. In the evaluation interval, the negative sample probability takes the value [0,1], which satisfies the linear functional relationship. During inference, negative sample predictions with higher rankings in the index list can help the network to optimally distinguish difficult samples, so they are more important than negative sample predictions with lower rankings. The negative sample probability multiplied by the importance can be expressed as a negative sample weighting function.

$$w_{\mathrm{neg}}=\left\{\begin{array}{cc} 1\times s^{\gamma } & \mathrm{IoU}<0.5\\ \left(-k\times {\mathrm{IoU}}^{\beta }+b\;\right)\times s^{\gamma } & 0.5<\mathrm{IoU}<0.95\\ 0 & \mathrm{IoU}>0.95 \end{array}\right.$$ (8)

where *γ* is the modulation factor. The *k* and *b* are the coefficients of the linear equation.

The design of the weighting function of positive and negative samples can distinguish between important and non-important samples. The method dynamically assigns a separate loss weight for positive samples and loss weight for negative samples to detection boxes, which is highly compatible with evaluation metrics.

#### Loss function

The loss function reflects the error size between the predicted value and the real value of the model in this paper, which is helpful to the iterative optimization in the process of model training and to evaluate the effectiveness of the model to the detection fruit. The model loss in the object detector consists of a combination of fruit classification loss and positive sample prediction bounding box bias loss.

$$L_{\text{detection}}=L_{\mathrm{cls}}+\lambda L_{\mathrm{reg}}$$ (9)

$$\begin{array}{c} L_{\mathrm{cls}}=\sum_{n=1}^{N}-w_{\mathrm{pos}}^{n}\times \ln\left(s^{n}\right)-w_{\mathrm{neg}}^{n}\times \ln\left(1-s^{n}\right)+\\ \sum_{m=1}^{M}\mathrm{FL}\left(s^{m},0\right) \end{array}$$ (10)

$$L_{\mathrm{reg}}=\sum_{n=1}^{N}w_{\mathrm{pos}}^{n}\times \text{GIoU}\left(b,b^{\prime }\right)$$ (11)

The loss *L*_detection_ of the FCOS-LSC is composed of *L*_cls_ and *L*_reg_. Here, *L*_cls_ denotes the predicted fruit category loss, *L*_reg_ represents the predicted regression loss, and *λ* is the modulation factor. *N* and *M* represent the number of detected frames in the candidate set and the number of detected boxes outside the candidate set, respectively. FL stands for focal loss [39]. GIoU is the regression GIoU loss [40]. *b*, *b′* are the positions of the predicted box and the real box.

Equations regarding FL in Eq. 10 and GIoU in Eq. 11 are shown below.

$$\begin{array}{c} \mathrm{FL}=\;\left\{\begin{array}{cc} -\alpha \times {\left(1-s^{m}\right)}^{\eta }\times \log s^{m} & s^{m}=1\\ -\alpha \left(1-\alpha \right){\left[1-\left(1-s^{m}\right)\right]}^{\eta }\times \log\;\left(1-s^{m}\right) & \text{otherwise} \end{array}\right. \end{array}$$ (12)

$$\begin{array}{c} {\text{GIoU}}_{\text{loss}}=1-\text{GIoU}\left(b,b^{\prime }\right)\\ \;=\;1-\left[\mathrm{IoU}-\frac{{\text{Bbox}}_{\min}-\text{Uion}\left(b,b^{\prime }\right)}{{\text{Bbox}}_{\min}}\right] \end{array}$$ (13)

Here, *α* is responsible for balancing the importance between positive and negative samples, and *η* is responsible for regulating the rate of weight reduction for simple samples. *Bbox*_min_ is the smallest enclosing convex object of *b* and *b^′^*.

## Results

In this paper, abundant experiments are conducted to verify the effectiveness of the optimized model for fruit detection. This section first introduces the experimental environment and the implementation details of the model during the training and testing periods. Then, the network is trained with the apple training dataset and the persimmon training dataset. The optimal training model is selected for testing on 2 validation datasets and for analyzing the results. Finally, state-of-the-art object detection algorithms are selected for experimental comparison in the same environment, and the results are analyzed and compared to verify the performance differences of the models in this paper in terms of fruit detection.

### Experimental settings

Experiments run on Ubuntu 18.04 64-bit operating system, 24 GB GTX 3090 graphics card, and 11.3 CUDA environment. All models use Python 3.7 version and Pytorch 1.11 version and build model components with the help of MMDetection 2.22.0 version learning library.

#### Image preprocessing

ResNet50 is used as the backbone network to extract fruit image features and then is inputted to the FPN for feature fusion. The fused features are features learned in 3 dimensions—scale, space, and channel—to obtain better information representation. The output 5 feature layers are all in 256 channel dimensions. The operation of the detection head is performed at each level, and its parameters are shared among each level. The final detection head outputs the prediction results for fruit category confidence and bounding box regression. Image preprocessing operations are performed before network training. First, the image is resized to a uniform scale. Next, the image is flipped with a random inversion probability of 0.5, then regularized, and finally padded to be divisible by 32 in downsampling. Image enhancement of the dataset prevents overfitting of the model due to insufficient data and enhances the generalization ability of the model.

#### Training

**Fig. 6.**
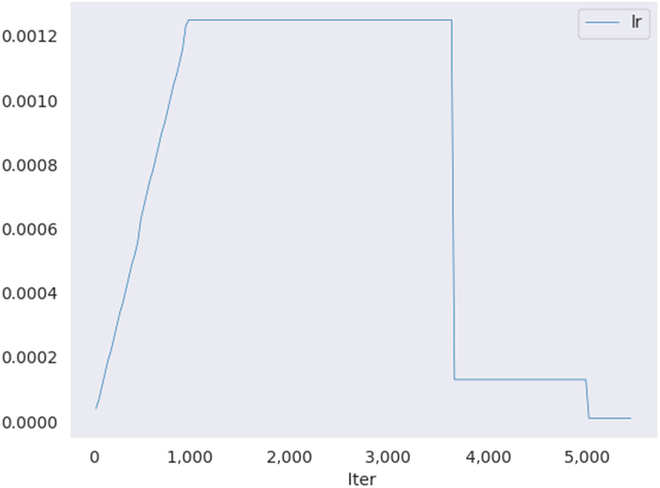
Learning rate change during training.

The learning rate for model training is set to 0.00125, the weight decay rate is set to 0.0001, and the momentum factor is set to 0.9. In this paper, the mini-batch method is used for training iterations for 12 epochs. The batch size per iteration is set to 2 fruit images, so the maximum number of iterations is 5,736. To prevent the gradient explosion during model training, the learning rate is adjusted using the warm-up strategy. The initial learning rate is adjusted linearly, i.e., the learning rate of the model increases linearly from 0.001 to 0.00125 in the first 1,000 iterations. The gradient is updated using the stochastic gradient descent optimizer (SGD) [41], and then the learning rate transformation is adjusted according to the number of iterations, that is, at the 8th epoch and 11th epoch of the iteration, it is reduced respectively to 1/10 of the original. The transformation of the learning rate is shown in Fig. 6.

**Fig. 7.**
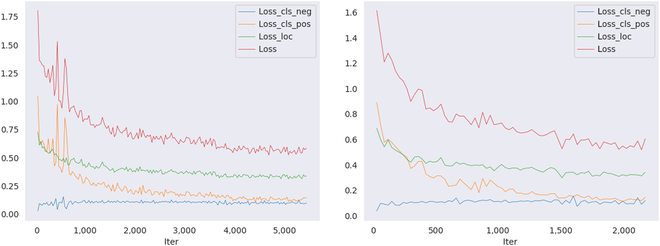
Changes in loss on 2 datasets in the model training phase. The left image is the apple dataset, and the right image is the persimmon dataset.

Using the above training parameters, the model profiles are set up in this paper to obtain the training loss variation curves on the apple dataset and the persimmon dataset, as shown in Fig. 7.

#### Testing

The same data preprocessing operations are performed before the images are input to the network, such as image cropping, random inversion, regularization, and padding. After the network prediction is over, the lower predicted values are filtered by setting a fruit confidence threshold of 0.4. The network then outputs the top 1,000 detection boxes with high confidence for each prediction layer. The network filters overlapping detection boxes by non-maximum suppression. The filtered detection boxes are still sorted by confidence. Each fruit image retains, at most, the first 100 confidence prediction boxes.

**Fig. 8.**
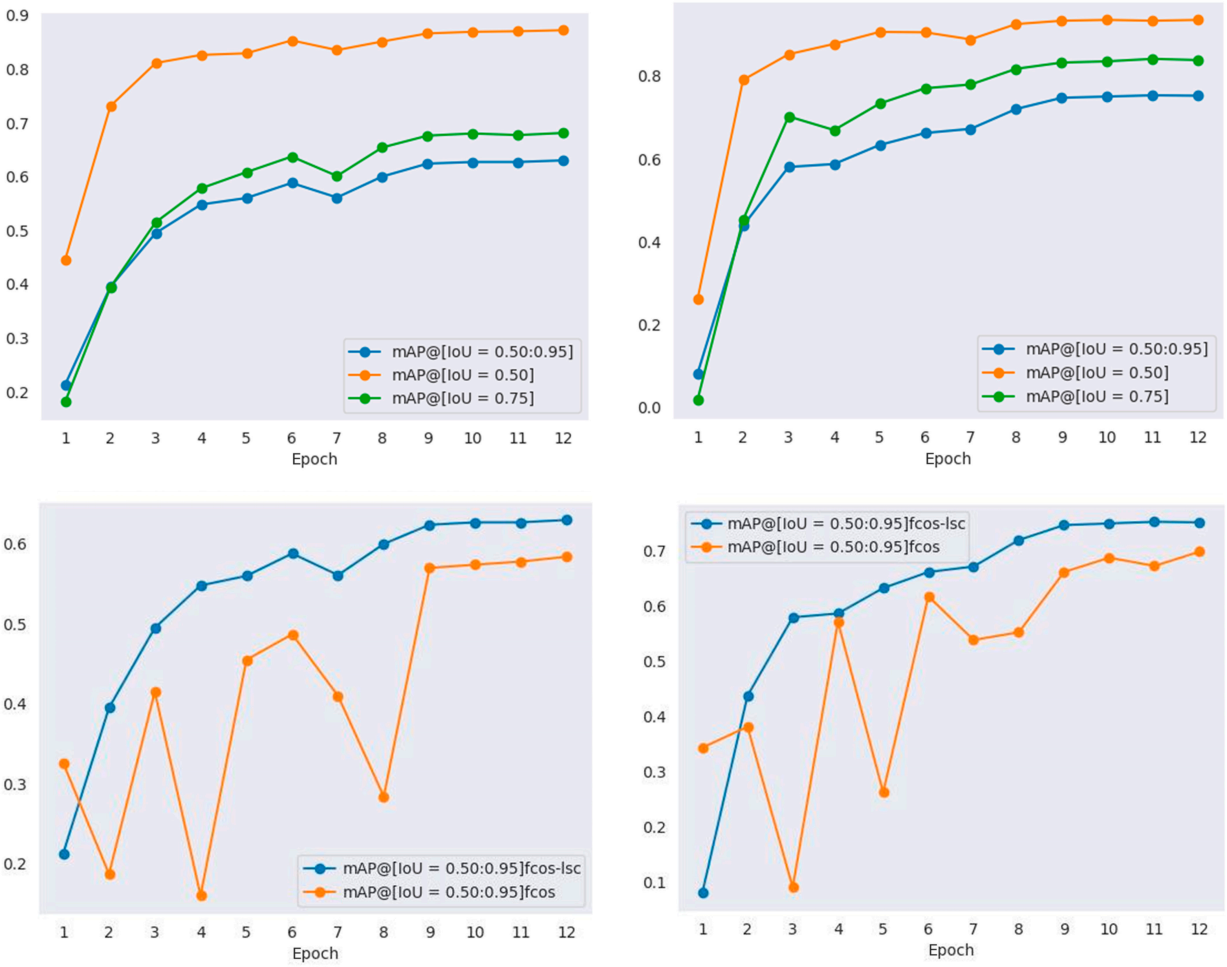
AP values under each epoch on the 2 datasets. (Left) Apple validation set. (Right) Persimmon validation set. The top row shows the change curves of AP values of FCOS-LSC on the 2 datasets, and the bottom row shows the change curves of AP values of FCOS-LSC compared with the baseline model on the 2 datasets.

The models with the above test parameters are used to validate the fruit images of the apple dataset and the persimmon dataset. The change curve of average precision (AP) obtained is shown in Fig. 8.

### Evaluation metrics

To better evaluate the FCOS-LSC model, this paper uses the AP and average recall (AR) under the IoU threshold of [0.5:0.05:0.95] to evaluate the performance of the model on fruit detection, where precision and recall can be expressed as the following formulas:

$$\text{Precision}=\frac{\mathrm{TP}}{\mathrm{TP}+\mathrm{FP}}\times 100\%$$ (14)

$$\text{Recall}=\frac{\mathrm{TP}}{\mathrm{TP}+\mathrm{FN}}\times 100\%$$ (15)

where TP is the number of fruits predicted as positive samples. FP is the number of backgrounds predicted as fruits, i.e., the number of false-positive samples. In addition, FN is the number of fruits not predicted as positive samples, i.e., the number of false-negative samples. Furthermore, the AP formula under a specific threshold can be obtained.

$${\mathrm{AP}}_{\mathrm{IoU}=i}=\frac{1}{101}\sum_{r\;\in \;\text{Recall}}\text{Precision}\left(r\right)$$ (16)

**Fig. 9.**
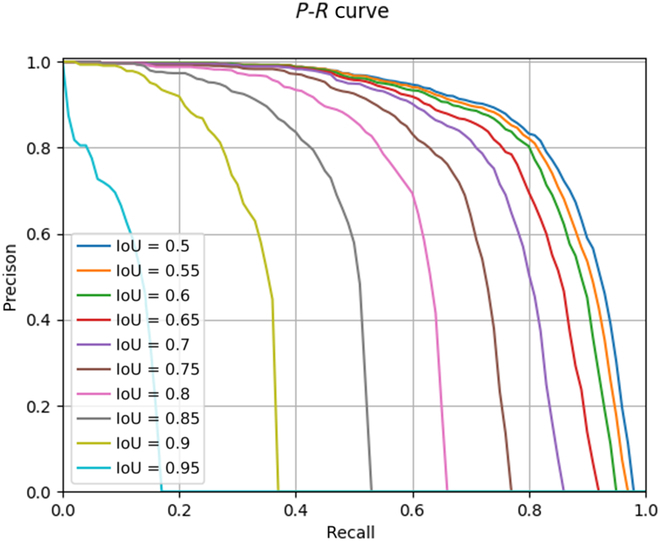
*P*–*R* curves at different thresholds on the apple dataset.

The AP at a certain threshold is obtained by taking the precision of 101 recalls at [0, 0.01, 1] and averaging them. This paper selects the IoU thresholds in the range of [0.5:0.95] every 0.05, a total of 10 thresholds, and averages them to obtain the evaluation indicators AP and AR. We also counted the AP and AR values at specific thresholds and different scales of large, medium, and small as the evaluation metrics for this experiment. The PR curves at 10 different thresholds on the apple dataset are shown in Fig. 9. Parameters (Params) are used to examine the number of parameters contained in the model to measure the simplicity of the model; floating point operations (FLOPs) measure the computational complexity of the model.

**Fig. 10.**
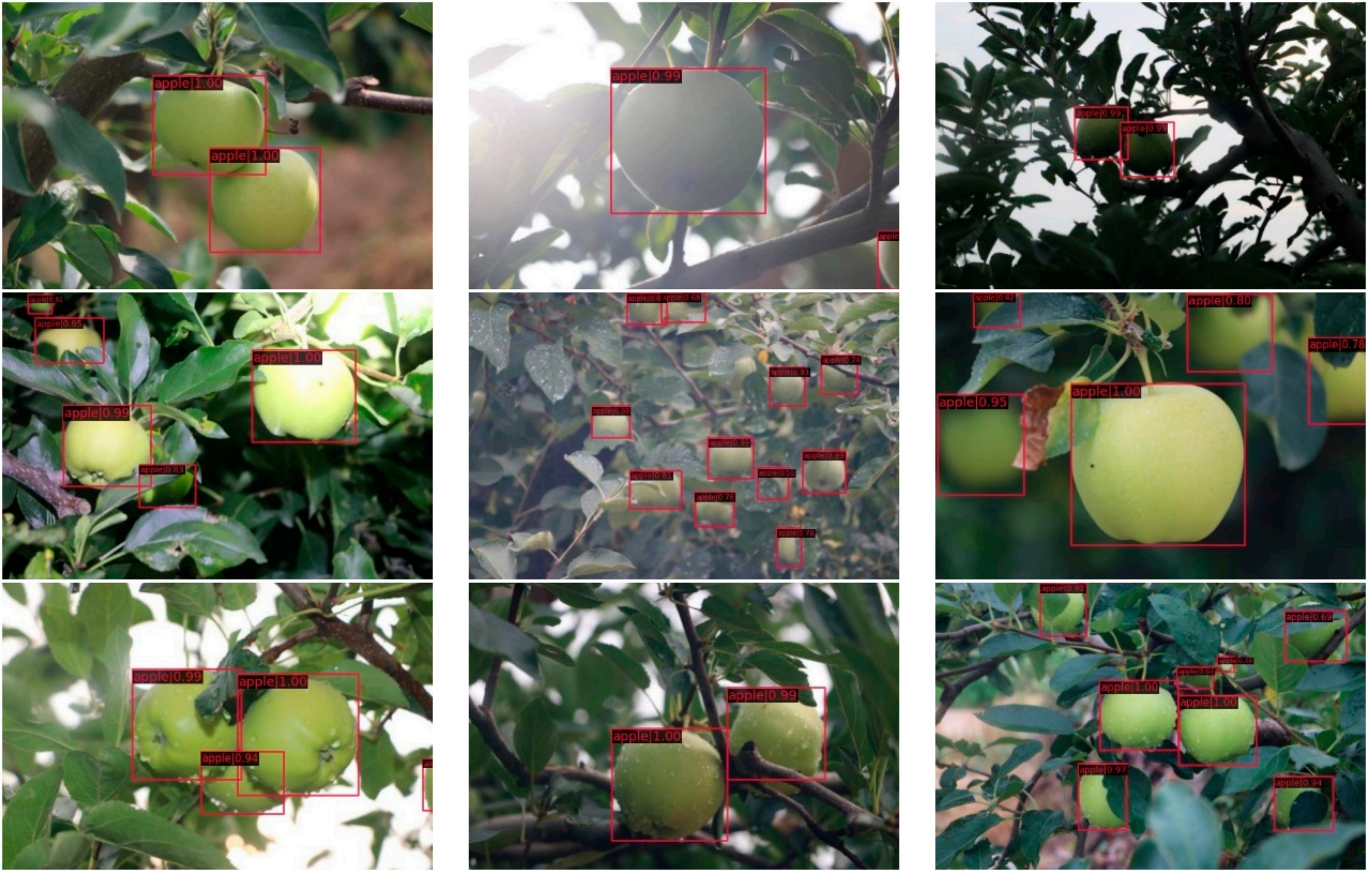
Apple dataset.

### Model detection effect

The proposed model is trained and tested on the apple dataset and the persimmon dataset, respectively. Figure 7 shows the visualization of all losses when our method is trained on the training set. The horizontal coordinates in the figure represent the number of iterations of the model during training, and the vertical coordinates represent the loss values. The red curve represents the total prediction loss of the model throughout the training period, including the negative sample weight loss change and positive sample weight loss change in classification loss. Negative and positive sample loss changes are presented by the blue and orange curves, respectively, as well as the green curve of regression loss change. The model optimizer SGD continuously optimizes until the model weight parameters converge during the training process. During each training epoch iteration, the test set is evaluated by the AR and AP.

**Fig. 11.**
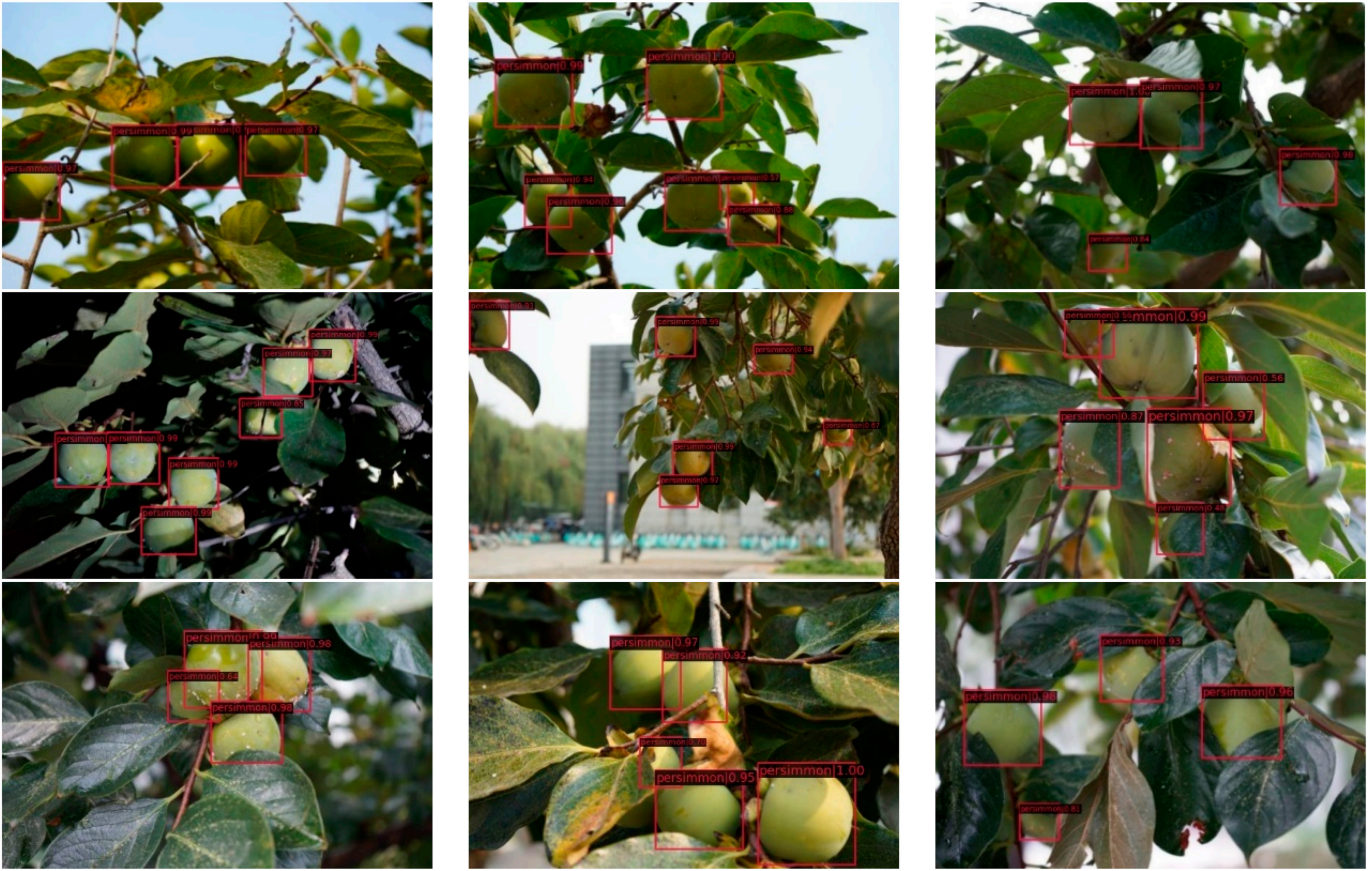
Persimmon dataset.

As shown in Fig. 8, the horizontal coordinate is the number of training iterations and the vertical coordinate is the prediction precision. In the first row, the AP values with thresholds of 0.75 and 0.5 for each epoch and AP with 10 thresholds within the interval are indicated by the green, orange, and blue curves. As shown in Tables 2 and 3, the method in this paper achieves 63% and 75% AP on the apple dataset and persimmon dataset, respectively. The second row of Fig. 8 compares the performance of the baseline model and FCOS-LSC on the green apple and green persimmon datasets, and the images show that FCOS-LSC is more stable during training and its performance is much better than that of the baseline.

In this paper, fruit images under mixed environments such as different lighting conditions, fruit occlusion, and distant view are selected for detection. The proposed method can accurately detect randomly taken fruit images with almost no misses and false detections, realizing a high detection accuracy. Acceptable results are achieved even when fruit images are mixed with background images that are not easily distinguishable, especially in the 2 cases of backlighting and blurred distant fruit. The model can also output detection results in a friendly manner when there are severe fruit overlaps and branch occlusions in the captured images, whose fruit contours are not clear. It can be seen that the model in this paper can perform the detection task accurately even with the occurrence of mixed interference conditions of various overlapping occlusions, lighting conditions, and shooting angle distances for fruit detection. Thus, the model based on improved FCOS is competent for the task of green fruit detection in orchards.

### Ablation experiment

To verify the effect of the LSC attention module and the positive and negative sample judgment methods, this paper further verifies the effectiveness of the 2 methods through ablation experiments. To understand the contribution of the 2 methods to the model, the new positive and negative sample selection method constructed is applied to the base model for a before-and-after comparison of apple detection effects, and the LSC module is added to further compare the effects. The experimental results are shown in Table 1.

**Table 1.** Validation of the 2 methods on the apple dataset. Input size: (600,400).

Model	AP	Params/M	FLOPs/GFLOPs
Baseline	58.4	31.84	48.62
+New sample selection method	61.8 (+3.4)	31.91 (+0.07)	49.90 (+1.28)
+LSC attention module	62.8 (+1.0)	38.65 (+6.74)	38.72 (−11.18)

First, on the basis of the original FCOS network, the positive and negative sample determination method is applied to the detection head, under the condition that the model parameters and computational complexity are the nearly same as the original model. The LSC module is then added to this, and after adding only a small number of parameters, the precision is improved by 1.0 percentage point and the complexity of the model is reduced. Therefore, the positive and negative sample determination method can better improve the ability to distinguish between green fruits and complex green backgrounds during training. Meanwhile, the LSC module enhances the ability of the model to represent features. The results are shown in Table 1. Although a small number of model parameters is added, the combination of the 2 methods results in an AP 4.4 percentage point higher than the original mode.

### Comparisons

To further analyze the effectiveness of this model in the implementation of fruit detection tasks, this paper selects the most advanced object detection algorithms for comparison. The comparison models include 2-stage anchor-based algorithms Faster R-CNN, Mask R-CNN, and its variant MS R-CNN, as well as one-stage anchor-based algorithms RetinaNet, YOLOv3, and ATSS, where ATSS is also compared as a label assignment policy method. Compared with the one-stage FCOS algorithm without anchor boxes, there is also the FoveaBox algorithm. All models are trained and tested on the apple dataset and persimmon dataset. The detection effect of each model is shown in Tables 2 and 3.

**Table 2.** Comparison of algorithms on the apple dataset. The best-performing experimental data in the table are represented by bold numbers.

Method	Backbone + Neck	AP	AP_0.5_	AP_0.75_	AP*_s_*	AP*_m_*	AP*_l_*	AR	AR*_s_*	AR*_m_*	AR*_l_*
Two-stage anchor-based											
Faster R-CNN	ResNet50 + FPN	59.6	85.9	65.8	43.6	67.0	84.3	65.5	51.5	72.4	88.3
Mask R-CNN	ResNet50 + FPN	60.1	86.3	66.5	44.9	67.4	84.9	66.4	53.2	73.0	88.2
MS R-CNN	ResNet50 + FPN	60.2	86.3	67.3	45.3	67.1	84.9	66.4	53.6	72.6	87.9
One-stage anchor-based											
RetinaNet	ResNet50 + FPN	57.6	84.9	62.6	42.2	65.1	82.8	65.1	50.8	72.6	87.5
YOLOv3	DarkNet53	59.4	84.6	65.2	40.8	65.9	87.5	65.9	51.6	71.9	91.7
ATSS	ResNet50 + FPN	62.1	**87.9**	64.7	46.1	67.2	88.8	69.3	56.4	75.0	92.0
One-stage anchor-free											
FoveaBox	ResNet50 + FPN	58.6	86.2	63.8	43.8	63.6	83.5	66.6	54.7	72.0	87.2
FCOS	ResNet50 + FPN	58.4	86.8	63.4	42.6	64.3	83.7	65.6	51.7	72.9	87.9
Ours											
FCOS-LSC	ResNet50 + FPN + LSC	**63.0**	87.2	**68.1**	**47.1**	**69.5**	**89.9**	**71.3**	**58.5**	**77.3**	**92.7**

**Table 3.** Comparison of algorithms on the persimmon dataset.

Method	Backbone + Neck	AP	AP_0.5_	AP_0.75_	AP*_s_*	AP*_m_*	AP*_l_*	AR	AR*_s_*	AR*_m_*	AR*_l_*
Two-stage anchor-based											
Faster R-CNN	ResNet50 + FPN	70.7	91.2	81.4	33.3	72.3	83.6	76.1	41.3	78.2	87.1
Mask R-CNN	ResNet50 + FPN	72.0	91.9	82.4	35.4	73.7	85.4	77.2	45.8	78.7	88.9
MS R-CNN	ResNet50 + FPN	73.1	92.1	83.7	34.9	75.1	85.9	77.9	48.5	79.3	88.8
One-stage anchor-based											
RetinaNet	ResNet5 0 +FPN	65.4	88.8	76.6	22.4	68.6	77.9	72.3	34.5	74.8	83.8
YOLOv3	DarkNet53	70.3	87.2	79.2	29.7	71.3	86.4	75.7	40.3	77.0	90.5
ATSS	ResNet50 + FPN	73.5	92.5	**84.1**	**38.5**	73.9	87.2	80.3	**55.2**	81.2	91.2
One-stage anchor-free											
FoveaBox	ResNet50 + FPN	69.6	91.3	80.0	30.2	71.4	81.8	76.4	46.0	78.1	86.6
FCOS	ResNet50 + FPN	69.9	91.9	79.3	34.7	71.4	82.3	76.7	48.4	77.9	87.6
Ours											
FCOS-LSC	ResNet50 + FPN + LSC	**75.2**	**93.5**	83.8	32.1	**76.7**	**89.1**	**80.9**	51.3	**82.2**	**92.4**

It is observed that, compared with other algorithms, FCOS-LSC has strong competitiveness in the performance of each evaluation metric on both the apple and the persimmon datasets. Despite considering the accuracy of model detection, it is also necessary to examine the capacity and computational complexity of the algorithm to balance the quality of the model design. Under the premise that the input image size is uniformly 600 × 400, each detection model capacity and complexity are calculated as shown in Table 4. Although FCOS-LSC is slightly inferior to ATSS in AP_0.5_ in the apple dataset and AP_0.75_ in the persimmon dataset as well as AP*_s_*, its algorithm complexity is reduced by 11.07G compared to ATSS. Compared with the model capacity and computational complexity of other algorithms, FCOS-LSC has the lowest computational complexity after introducing a small number of model parameters.

**Table 4.** Comparison of the number of parameters and flops computational complexity of models. Input size: (600,400).

Method	Params/M	FLOPs/GFLOPs
Two-stage anchor-based		
Faster R-CNN	41.12	61.52
Mask R-CNN	43.75	113.0
MS R-CNN	60.01	113.0
One-stage anchor-based		
RetinaNet	36.10	50.55
YOLOv3	61.52	47.88
ATSS	31.89	49.79
One-stage anchor-free		
FCOS	31.84	48.62
FoveaBox	36.01	50.06
Ours		
FCOS-LSC	38.65	**38.72**

**Fig. 12.**
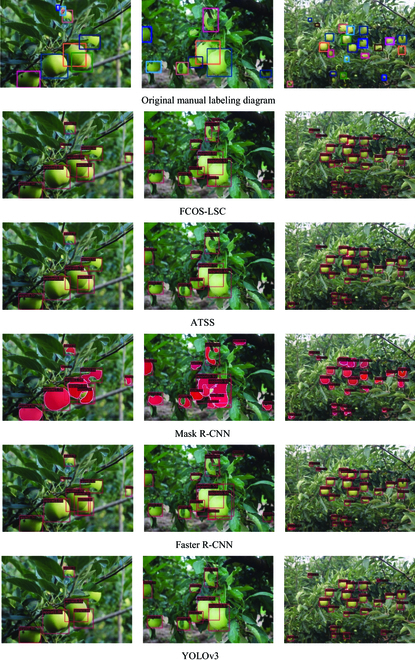
Detection results of different algorithms on the apple dataset.

**Fig. 13.**
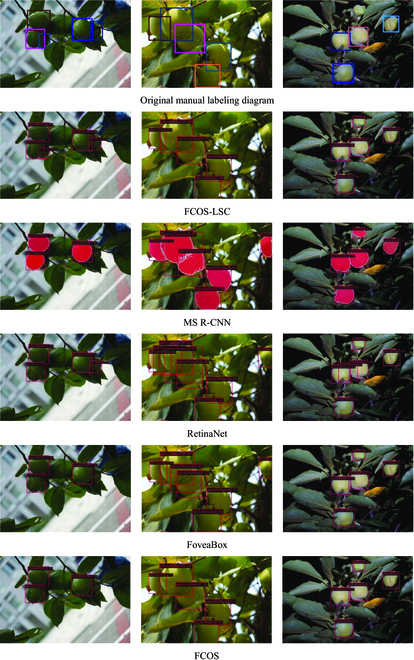
Detection effect of different algorithms on the persimmon dataset.

In this paper, Faster R-CNN, Mask R-CNN, YOLOv3, and ATSS algorithms are selected for the detection of fruit images in the apple dataset, as shown in Fig. 12. MS R-CNN, RetinaNet, FoveaBox, and FCOS algorithms are selected for the detection of fruit images in the persimmon dataset, as shown in Fig. 13. From the figure, it is easy to find that the fruits with clear fruit contours can be detected and have the highest detection accuracy performance. At the same time, fruits with blurred edges and even unlabeled fruits can be detected accurately, as shown in the first image of the apple dataset. This is very helpful to deal with the situation of fruit detection in complex orchards with multiple interference factors.

In summary, the FCOS-LSC model is more concise in design, has fewer requirements on memory and calculation, and realizes the prediction of higher probability values, which can meet the real-time orchard operation tasks and present more comprehensive and efficient results.

### Failure detections

**Fig. 14.**
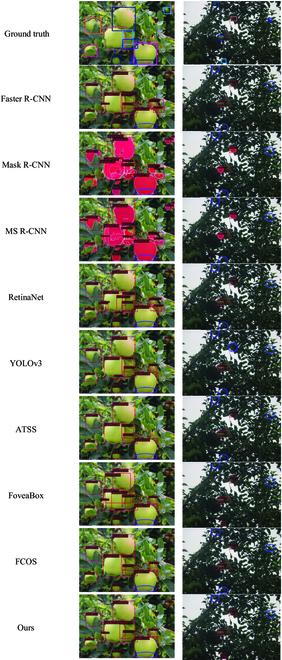
The visualization of all the models mentioned in this paper on the apple dataset.

This section further analyzes the difficult problems of the existing detection tasks in orchards. To better illustrate the detection effectiveness of FCOS-LSC, we chose fruits with a heavy overlap in the orchard and fruits under a distant and backlight condition. The visualization of all the models mentioned in this paper on the apple dataset is presented as shown in Fig. 14. FCOS-LSC can still accurately detect the target fruit in the presence of missed detection by other comparison models. However, not all target fruits can be detected, as detailed in the marked section. The model misses the obscured target fruits in the close-up images because of the severe shape deficit, and this is also a common problem with other models. In addition, when dealing with backlight images, the model does not work well because of factors such as the small size of the fruit and the presence of occlusion.

### Discussion

In previous studies, a combination of deep learning techniques and image processing has made significant progress in target fruit recognition tasks [42]. The 2-stage detection model has high detection accuracy relative to the 1-stage model but involves the design of the anchor frame, and the complexity and computational volume of its model increase along with it. Considering the needs of orchard robot operations, designing algorithms with high detection accuracy and low computational cost is the key to coping with the target fruit identification and localization problem. Object detection task embedding visual attention during model training is an effective way [43–46] to focus on some of the input features instead of the whole input for solving the target task. In addition, to improve the generalization ability and robustness of the model, it is crucial to design a more reasonable positive and negative sample selection strategy [47,48].

Experimental results show that the proposed model achieves better accuracy with relatively fewer parameters and fewer FLOPs. The proposed FCOS-LSC model outperforms other state-of-the-art algorithms in terms of detection accuracy and efficiency. As shown in Tables 1 and 4, FCOS-LSC exhibits high AP, and the analysis reveals that the model does not have its model computational complexity enhanced by the addition of the LAC module but instead has the least FLOPs, which is due to the dimensional adjustment of the feature map that promotes the model to focus more on effective features, as described in detail in the “LSC attention module” section. Although FCOS-LSC performs well in the visualization effect map, it is relatively poor in small target fruits, as shown in Tables 2 and 3, where the enhancement effect of model optimization shows AP_l_ > AP*_m_* > AP*_s_*. This phenomenon is caused by the positive and negative sample selection strategy. The method first selects the detection frame with the true bounding box near the center point to better discriminate the supervised signal, which may lead the method to be more focused on important samples compared to samples near the target fruit boundary, while this method is friendly to large targets. In conclusion, the high accuracy and robustness of the model provide further possibilities for its deployment with intelligent agricultural equipment, which can meet the needs of real-time operations

## Conclusion

The purpose of this study is to deploy and apply the technology of green fruit detection to agricultural intelligent equipment to meet the task of fruit identification in complex orchards. The one-stage anchor-free FCOS model is optimized to avoid the reliance on anchors in the fruit detection process, thus shortening the detection time, which can be widely applied to other agricultural fields. The deformable convolution is added in the backbone network to better adapt to the green fruit target with different shapes. The convolution-based attention operation is applied to the fused features, which combines low-level detail information and high-level semantic information to improve the scale, space, and channel feature representation of the features. Moreover, this operation helps the network deal with overlapping occluded fruits to achieve better detection results. To distinguish the green target fruit from the background, a new sample selection strategy is constructed to provide more discriminable supervised signals by specifying loss weights for positive and negative samples and applying them directly to the detection head.

Although the FCOS-LSC model achieves better detection results on green apple and persimmon datasets, there is still space for improvement. More types of green fruit images are collected to verify the effectiveness of the model, and the model is designed to improve the detection of small target fruits. In the practical application of unstructured orchards, the accuracy and time efficiency of the model need to be considered, so the model will be further optimized to improve the overall efficiency of the model by shortening the computation time while improving the detection accuracy.

## Data Availability

Anyone who wants to use the data can contact the corresponding author W.J. The author is with the School of Information Science and Engineering, Shandong Normal University, Jinan 250358, China (e-mail: jwk_1982@163.com).
